# High Intensity Jump Exercise Preserves Posture Control, Gait, and Functional Mobility During 60 Days of Bed-Rest: An RCT Including 90 Days of Follow-Up

**DOI:** 10.3389/fphys.2018.01713

**Published:** 2018-12-03

**Authors:** Ramona Ritzmann, Kathrin Freyler, Jakob Kümmel, Markus Gruber, Daniel L. Belavy, Dieter Felsenberg, Albert Gollhofer, Andreas Kramer, Gabriele Ambrecht

**Affiliations:** ^1^Institute of Sport and Sport Science, University of Freiburg, Freiburg, Germany; ^2^Sensorimotor Performance Lab and Human Performance Research Centre, University of Konstanz, Konstanz, Germany; ^3^Centre of Muscle and Bone Research, Charité University Medicine Berlin, Berlin, Germany; ^4^Institute for Physical Activity and Nutrition, School of Exercise and Nutrition Sciences, Deakin University, Geelong, VIC, Australia

**Keywords:** balance, locomotion, countermeasure, neuromuscular, deconditioning, chair rising, timed up and go

## Abstract

Physical inactivity causes a deconditioning of the human body. Concerns due to chronic bed-rest include deficits in posture and gait control, predisposing individuals to an increased fall and injury risk. This study assessed the efficiency of a high-load jump exercise (JUMP) as a countermeasure to prevent detrimental effects on gait, posture control and functional mobility. In an RCT (23 males), the effect of 60 days bed-rest without training was compared to JUMP. JUMP is characterized by plyometric executed as a high intensity interval training. Typical trainings session consisted of 4 × 10 countermovement jumps and 2 × 10 hops in a sledge jump system. We assessed sway path and muscle activity in monopedal stance, spatiotemporal, kinematic, and variability characteristics in gait, functional mobility with repeated chair-rises and Timed Up and Go (TUG). Results revealed: The JUMP group showed no significant changes after bed-rest, whereas the control group exhibited substantial deteriorations: an increased sway path (+104%, *p* < 0.05) was accompanied by increased co-contractions of antagonistic muscles encompassing the ankle (+32%, *p* < 0.05) and knee joint (45%, *p* < 0.05). A reduced locomotor speed (−22%, *p* < 0.05) was found concomitant with pathological gait rhythmicity (*p* < 0.05), reduced joint excursions (ankle −8%, knee −29%, *p* < 0.05) and an increased gait variability (*p* < 0.05). Chair-rising was slowed (+28%, *p* < 0.05) with reduced peak power (+18%, *p* < 0.05), and more time was needed to accomplish TUG (+39%, *p* < 0.05). The effects persisted for a period of 1 month after bed-rest. Increases in sway path were correlated to decreases in gait speed. The JUMP effectively preserved the neuromuscular system's ability to safely control postural equilibrium and perform complex locomotor movements, including fast bipedal gait with turns and rises. We therefore recommend JUMP as an appropriate strategy combatting functional deconditioning.

## Introduction

Physical inactivity causes a progressive deconditioning of the human body (Blair, 2009). Deconditioning has serious structural and functional consequences: in addition to the loss of bone (LeBlanc et al., 2007) and muscle mass (Booth et al., 2012), deficits in posture control (Dupui et al., 1992; Kouzaki et al., 2007), locomotion (Dupui et al., 1992), and functional mobility (Gill et al., 2004; Reschke et al., 2009; Miller et al., 2018) have been found in response to disuse and aging, for instance during bed-rest (Pavy-Le Traon et al., 2007) or exposure to weightlessness during space flights (Adams et al., 2003). Significant adverse effects entail fragility, falls, fractures and an impaired quality of life (McGregor et al., 2014).

With a persistency beyond the acute period of inactivity followed by long recovery periods (Pavy-Le Traon et al., 2007), physical deconditioning has gained socio-economic importance in various scenarios, increasingly raising scientific debate about auspicious countermeasures (Booth et al., 2000). Relevant scenarios include a sedentary lifestyle over the lifespan, particularly in the presence of age (McGregor et al., 2014), disease (Booth et al., 2000) or disability (Booth et al., 2000). Particularly for the elderly (McGregor et al., 2014), but also for ill or bed-ridden patients (Pavy-Le Traon et al., 2007), physical deconditioning became a substantial problem in a contemporary society that cultivates a modern inactive lifestyle (Booth et al., 2000). The cumulative effect of muscle weakness in the lower extremities, coupled with postural and locomotor instability, have especially been identified as increasing the incidence of injury in individuals with a compromised integrity of the neural and skeletal system (Campbell et al., 1989; Rubenstein, 2006). This is associated with the high clinical and consequential costs related to reduced autonomy and increased care needs in response to functional deconditioning as documented by epidemiological studies (Booth et al., 2000; Rubenstein, 2006).

Regular physical exercise has been identified as advantageous as a countermeasure against functional deconditioning, above nutrition or pharmacological treatments (Booth et al., 2000; Trappe et al., 2007; Viguier et al., 2009; Gast et al., 2012). Its efficacy has been reported consistently. Amongst the great diversity of physical exercises, a small selection of simple, purposive and time-efficient modalities have come into focus (Rittweger et al., 2006; Belavý et al., 2010, 2017; Kramer et al., 2017a,b, 2018). Distinguished by a high compliance and marked effects on the preservation of the bone, muscle mass and function of the leg and the cardiovascular system, short-term plyometric jump exercise has been validated an RCT as an efficient countermeasure for muscle and bone damage requiring a small daily effort of about 3 min (Kramer et al., 2017a,b, 2018). Plyometric jumps are complex full-body movements characterized by a notably high peak force and peak power which require maximal physical effort (Taube et al., 2012). The muscle action relies on the stretch-shortening cycle which is distinctive for the class of locomotor movements and based on a complex motor pattern (Taube et al., 2012). These exercise attributes collectively justify the expectation that plyometric jumps may be a promising intervention for preserving daily movement skills with high coordinative demand. The benefits for musculoskeletal structures have been clearly outlined, but the effect on the functional skills relevant for daily life, including a safe posture, and gait control entailing functional mobility related to standing, sitting, moving, and turning, have not yet been established. Relying on sensorimotor control, those factors are highly correlated to falls (Tinetti et al., 1988), and associated deficits show high prevalence rates for momentous injuries and hospitalization (Salgado et al., 1994; Rubenstein, 2006). A functional evaluation is required to make a conclusive statement about the efficiency of this countermeasure. Neuromuscular investigations furthermore may help to assess the mechanisms underlying the functional degradations, and explain the potential benefit of the selected countermeasure.

Therefore, the objective of this study was to evaluate the efficacy of a high-intensity plyometric jump training as a countermeasure for the deconditioning effects of physical inactivity related to locomotion and posture control. With reference to the aforementioned findings (Dupui et al., 1992; Gill et al., 2004; Kouzaki et al., 2007; Reschke et al., 2009), we focused on the acute effects immediately after bed-rest and the 90 days recovery period. We hypothesized that the training group would significantly differ from a control group after 2 months of bed-rest with respect to the control of posture (with eyes open and eyes closed), locomotion (preferred and maximal gait speed), mobility (Timed Up and Go), and function of the lower limbs (chair-rising test). Thereby, we expected a significant loss of these movement skills in the control group along with a significantly shortened recovery period or even the complete preservation of these skills for the training group.

## Materials and methods

### Experimental design

**Figure 1:**
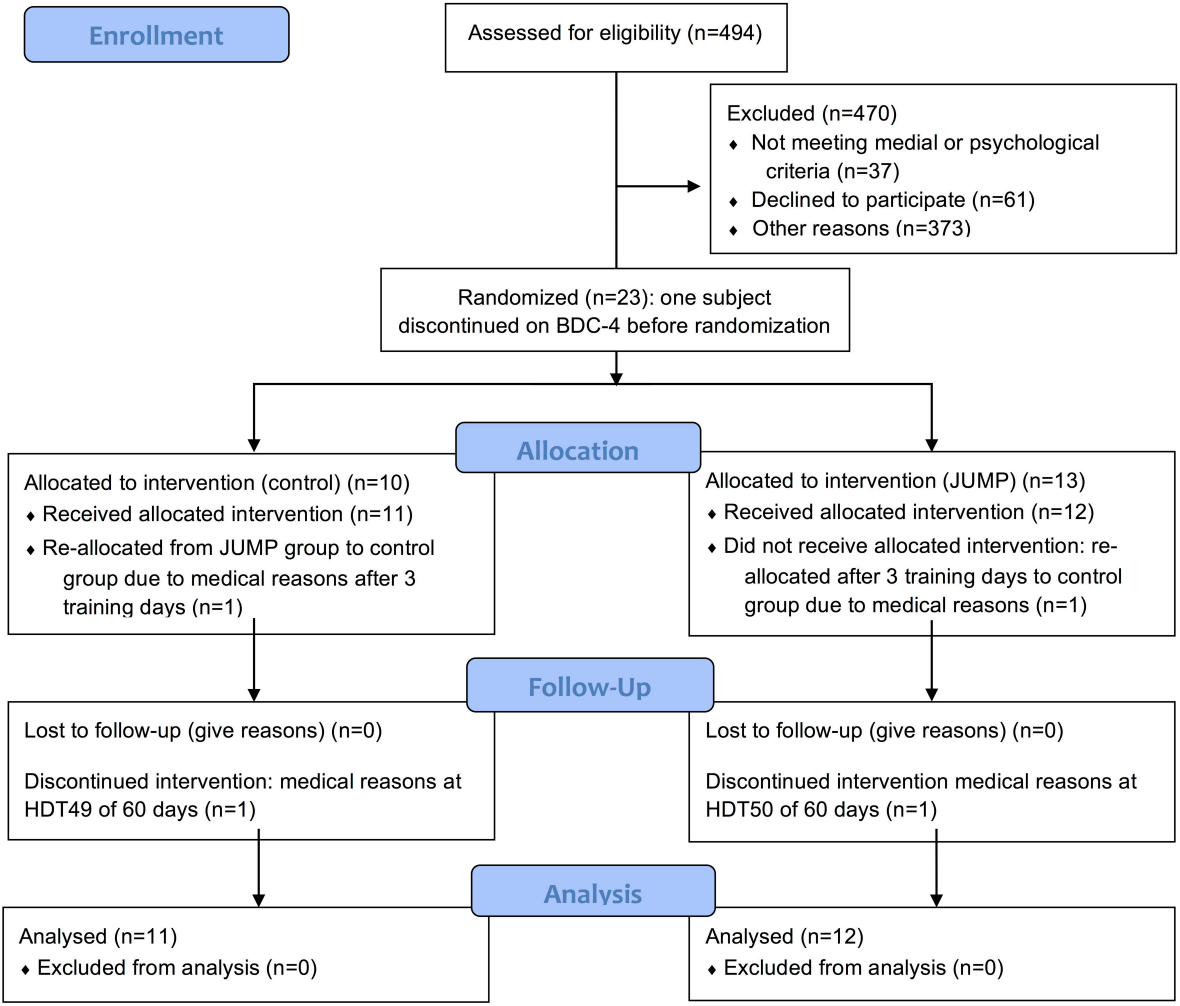
CONSORT Flow diagram of the progress through the phases of a parallel randomized trial of the CTRL and JUMP groups split into the enrolment, intervention allocation, follow-up, and data analysis from the top to the bottom.

This randomized controlled study was conducted at the German Aerospace Center (DLR, Cologne, Germany). The longitudinal study involved 15 days of familiarization, including baseline data collection (BDC), 60 days of 6° head-down tilt bed-rest for 24 h/day (HDT) and 90 days of recovery (Figure 1). Physical activity during the familiarization (BDC) and recovery phases was restricted to free movement in the facility, and reeducation training during the recovery phase. The control of posture, gait and functional mobility was tested for all subjects at seven time points: 1 day after arrival at the facility (BDC-14), 1 day before (BDC-1) and after bed-rest (R+0), as well as during recovery (R+7, R+13, R+28, and R+90). For details about the study design, schedule, diet, subject recruitment and eligibility criteria see Kramer et al. (2017b).

### Subjects

The 24 volunteers were selected from a large group of actively recruited males. A priori, the sample size was estimated by means of a power analysis based on the results of previous bed-rest studies (f = 0.4; alpha = 0.05; power = 0.9) with a margin of two dropouts (Dupui et al., 1992). The study was performed in two campaigns (autumn 2015 and spring 2016). Recruitment started approximately 6 months before the first campaign directly after approval from the ethics committee and was finalized early in 2016 with a total recruitment time of 8 months. Inclusion criteria were as follows: male, age between 20 and 45 years, body mass index between 20 and 26 kg/m^2^, non-smoking, no medication, no competitive athlete, no history of bone fractures and medical issues documented in detail in Kramer et al. (2017b). The participants gave written informed consent for the experimental procedure, which was approved by the ethics committee of the Northern Rhine Medical Association (No. 2014105, Dusseldorf, Germany) and the Federal Office for Radiation Protection (Berlin, Germany). The study was designed according to the most recent iteration of the Declaration of Helsinki. All subjects were in good health and were randomly allocated to either a jump exercise group (JUMP) or to a control group (CTRL) using dice roll of each pair of participants in the morning of the first day of HDT. The mean ± SD age, height and body mass were 30 ± 7 years, 181 ± 7 cm and 77 ± 7 kg for JUMP (*n* = 12) and 28 ± 6 years, 181 ± 5 cm and 76 ± 8 kg for CTRL (*n* = 11). Of the 24 healthy male subjects that were enrolled in the study, one subject discontinued the study on BDC-4 for medical reasons unrelated to the study. The subject could not be replacement due to time constrains leaving a total of 23 study participants. One participant started in the training group, but was reallocated to the control group after three training sessions due to a possible medial tibia stress syndrome. Two of the 23 subjects that completed the study (one CTRL, one JUMP) were re-ambulated after respectively, 49 and 50 instead of 60 days of HDT due to medical reasons, but completed the recovery phase with all the scheduled measurements.

### Countermeasure

While subjects in the CTRL group were inactive, subjects in the JUMP group participated in the training intervention during strict 6° head down tilt bed-rest for 60 days, executed under medical supervision. The subjects trained in a sledge jump system (Novotec Medical GmbH, Pforzheim, Germany) allowing natural jumps in the horizontal plane with different acceleration levels (Kramer et al., 2012). The training protocol for the JUMP group comprised 48 training sessions with an effective training duration of approximately 3 min; each session was varied, but contained on average 4 × 12 countermovement jumps and 2 × 15 repetitive hops (Figure 1). A warm-up prior to the training consisted of 6 squats, 6 heel raises, 3 submaximal countermovement jumps, and 10 submaximal hops. All sessions were supervised; peak forces and peak power were documented. The subjects underwent nine 30 min familiarization sessions during BDC to get accustomed to the device and develop the correct jumping technique. Further details about the countermeasure, training procedures and familiarization sessions can be found in Kramer et al. (2017b).

### Protocols

**Figure 2:**
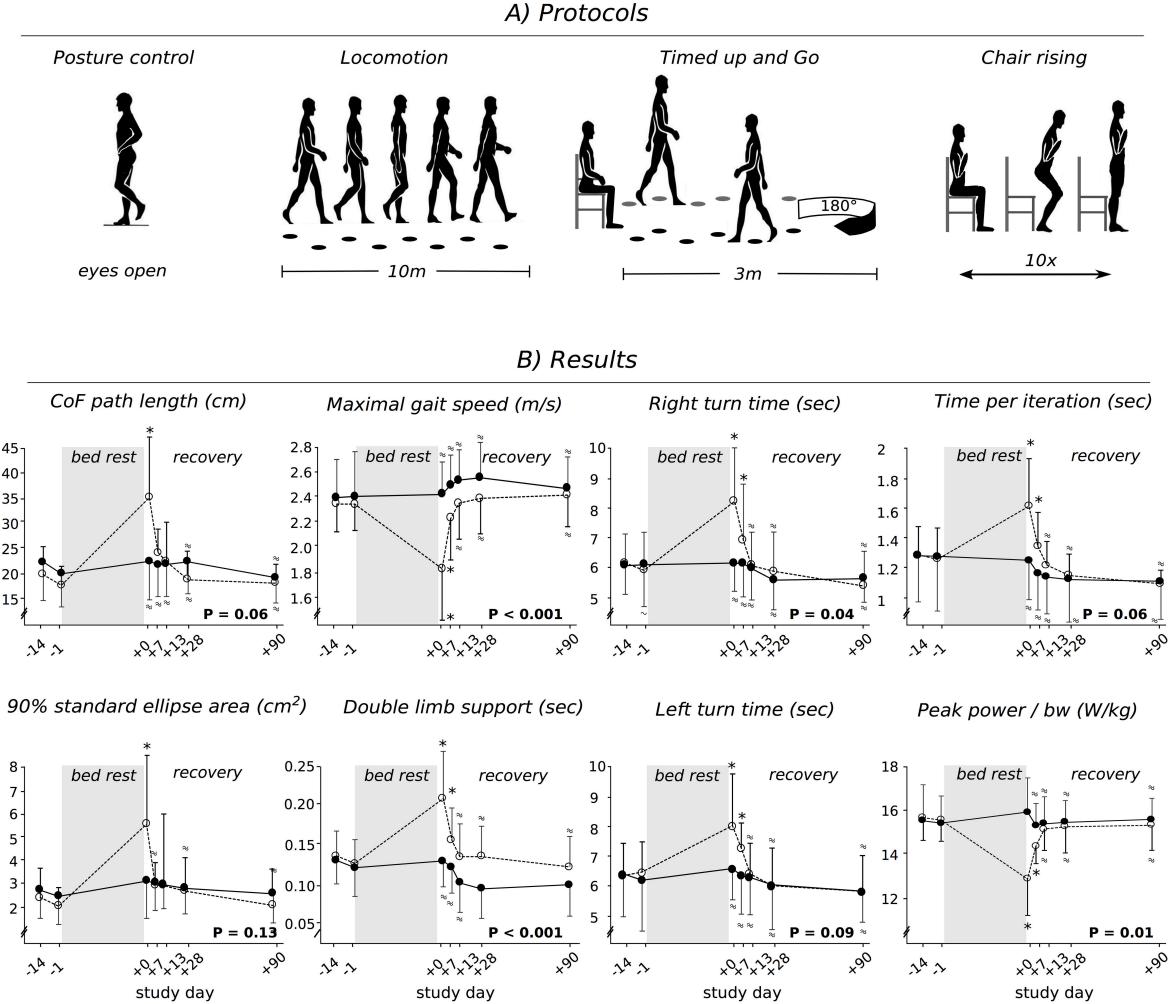
Protocol schematic **(A)** and grand means **(B)** for the inactive CTRL (open circles) and the JUMP group (full circles). Data is displayed for baseline measurements (BDC-14 and BDC-1) and the recovery period (R+0, R+7, R+13, R+28, and R+90) after the 60 days of bed-rest. Changes in center of force (CoF) path length and standard ellipse area in the balance test, gait speed and double limb support, time for the left and right turn for “Timed Up and Go” test, time and peak power chair-rising with respect to baseline (BDC-1). Values are means ± SE. *P* < 0.05 denotes a significant group^*^time interaction effect. ^*^ indicates a significant difference compared to baseline. If values were statistically non-inferior compared to baseline, they are marked with a ≈ symbol.

Four protocols served to assess changes in posture, gait control, and functional mobility in response to bed-rest (Figure 2A). They were executed in the same order for each subject, but were randomized among the participants (subjects and therapists were not blinded; assessors were blinded).

#### Posture control

The control of posture was assessed in the monopedal stance with eyes open (EO) and eyes closed (EC) on a force plate (Leonardo Mechanography®, Novotec, Pforzheim, Germany) according to Freyler et al. (2014) using the data acquisition unit Power1401-3 (CED, Cambridge, United Kingdom). The subjects stood barefoot in an upright position on their left leg, kept hands on their hips and directed their head and eyes forward. They were instructed to stand as still as possible, with the free leg not touching the other leg. Recordings were made twice in each condition over a period of 10 s, separated by 1 min breaks; means were calculated. We assessed the displacement and velocity of the center of force (CoF), the dominant frequency and the standard ellipse area (90% movement area) using MATLAB® R2016a. The CoF displacement and standard ellipse area in medio-lateral (ML) and anterior- posterior (AP) direction were calculated.

Monitoring of muscle activation was executed via surface electromyography (EMG). Wireless electrodes (Trigno, Delsys, USA) were placed over the left leg soleus (SOL), medial gastrocnemius (GM), tibialis anterior (TA), rectus femoris (RF), vastus lateralis (VL) and biceps femoris (BF) muscles, according to SENIAM (Hermens et al., 2000). The longitudinal axes of the electrodes were in line with the presumed direction of the underlying muscle fibers. Inter-electrode resistance was reduced by shaving and lightly abrading the skin. Signals were sampled with 2,000 Hz and band-pass filtered (20 to 450 Hz, effective signal gain of 909) using the data acquisition unit Power1401-3 (CED, Cambridge, United Kingdom). Pseudo isometric maximum voluntary contractions (pMVCs) were performed for normalization. PMVCs were executed once for all recorded muscles against manual resistance for 3 s with standardized knee and hip joint angles according to Freyler et al. (2016). To assess the simultaneous activation of antagonistic muscles encompassing the ankle and knee joint, the co-contraction index (CCI) was calculated for TA_SOL, GM_TA, BF_VL and BF_RF, with the rectified and pMVC-normalized EMG by means of the following equation: CCI_i_ = Σ (lower EMG_i_ / higher EMG_i_) × (lower EMG_i_ + higher EMG_i_) for each sample point, CCI = Σ CCI_i_ (Freyler et al., 2014).

#### Gait

**Figure 3:**
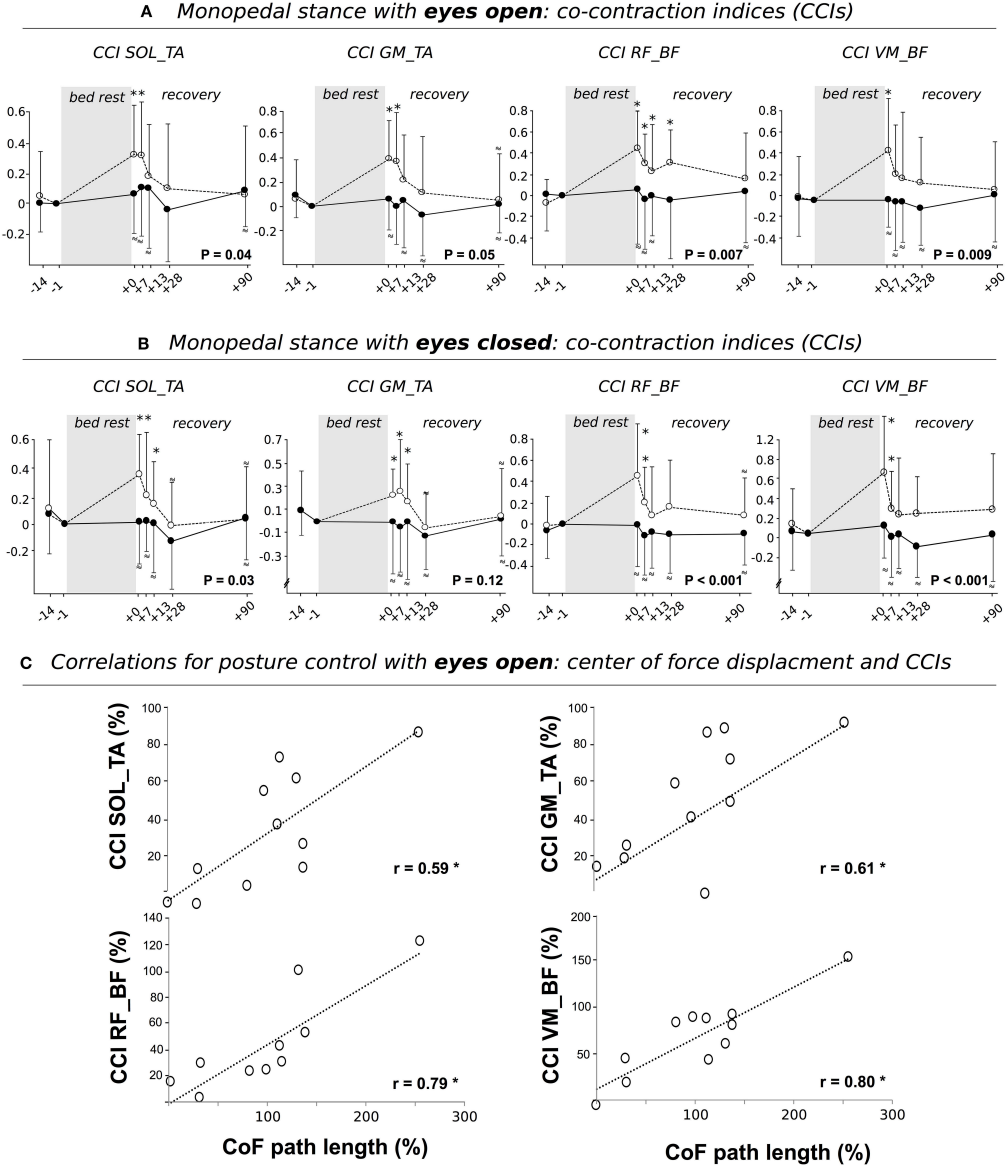
Grand means of the co-contraction index (CCI) during posture control performed with **(A)** eyes open and **(B)** eyes closed for the CTRL (open circles) and JUMP group (full circles). Data is displayed for baseline measurements (BDC-14 and BDC-1) and the recovery period (R+0, R+7, R+13, R+28, and R+90) after the 60 days of bed-rest. Results show changes in antagonistic muscle groups encompassing the ankle (m. soleus and tibialis (SOL_TA); m. gastrocnemius medialis (GM) and TA (GM_TA) and knee joint (m. rectus femoris and m. biceps femoris (RF_BF); m. vastus medialis and BF (VM_BF) indicating an increased CCI after bed-rest. *P* < 0.05 denotes a significant group^*^time interaction effect. ^*^ indicates a significant difference compared to baseline. If values were statistically non-inferior compared to baseline, they are marked with a ≈ symbol. **(C)** Pearson's correlation coefficients among the variables center of force (CoF) path length (abscissa) with CCIs for differences between values obtained at R+1 and BDC-1 for CTRL. Findings revealed positive correlations, indicating an interrelationship between the increased sway path and augmented antagonistic co-contraction. Values are means ± SE. ^*^indicates significant findings (*P* < 0.05).

Subjects performed a 10 m walk at their preferred and maximal gait speed. We used Optogait (Optogait; Microgate, Bolzano, Italy) and 2D kinematics (Panasoni, Simi Motion 2D, Simi Reality Motion Systems GmbH, Unterschleissheim, Deutschland) to assess group differences in the locomotor pattern with an emphasis on spatiotemporal, kinematic and variability characteristics (Figures 2, 3). The data was extracted at sampling frequencies of 1,000 and 200 Hz. Trials were repeated twice and averaged. To assess changes in gait biomechanics and to evaluate gait quality over time we calculated the total vertical center of mass movement per step, plantarflexion at push off, minimal foot clearance during the swing phase and maximal knee flexion during the swing phase from the 2D kinematics according to Böhm et al. (2014) and van der Linden et al. (2014). The extracted parameters are of clinical relevance, and correlated to an increased fall and injury predisposition (Kerrigan et al., 1995; Lai et al., 2012). Gait speed, step length and step time, cadence, double limb support and single limb support time with flat foot, stance phase, and swing phase expressed as a percentage of the total gait cycle time (GCT) were also assessed. The coefficient of variation (CV) was calculated for step length, step time and stance time, to estimate characteristics of gait (ir)regularities. Fluctuation in the spatiotemporal characteristics is a sensitive indicator addressing mobility deficits in the locomotor pattern (Gouelle and Megrot, 2018).

#### Timed up and go test (TUG)

The TUG—commonly used to examine mobility in community-dwelling or frail adults—was used to assess changes in functional mobility (Podsiadlo and Richardson, 1991). The test requires a subject to stand up from a chair, walk 3 m, turn, walk back, and sit down again (Figure 2A). The time taken to complete the test is strongly correlated to the level of functional mobility.

#### Chair-rising test (CRT)

The CRT (10 chair rises, Figure 2A) was used to measure power on vertical movement and muscle function surrounding the hip as the most important neuromuscular risk factor for falls and fall-related fractures (Alexander et al., 1991). Arms were crossed in front of the chest; full knee extension and buttock contact with the chair was controlled visually in all subjects and repetitions. Time per iteration (s) and maximal power in the rising phase normalized to bodyweight (W/kg) were assessed on a force plate with signals sampled with 2,000 Hz (Leonardo Mechanography®, Novotec, Pforzheim, Germany; Busche et al., 2013). Thereby, a phase of quiet sitting served to assess the body weight. Subsequently, the dynamic acceleration acting on the center of mass (COM) was calculated according to a = F/m and the vertical velocity of the COM was calculated as the integral of the dynamic acceleration over time. Power was calculated as the force multiplied by the velocity for each sample point (P = F^*^v). The peak power was then assessed during each rise phase of the CRT and subsequently averaged for the 10 repetitions.

### Statistics

A repeated measures analyses of variance (ANOVA), with time [BDC-1, R+0, R+7, R+13, R+28, R+90] as the repeated measure and group [JUMP vs. CTRL] as the inter-subject factor was used to test for adaptations in response to bed-rest. The normality of the data was evaluated with a Kolmogorov-Smirnov test; the data followed a normal distribution. If the assumption of sphericity established by Mauchly's test was violated, the Greenhouse-Geisser correction was used. The level of significance was set to *p* < 0.05. To compare each time point in the recovery period with BDC-1, Student's *t*-tests were used. The false discovery rate was controlled according to the Benjamini-Hochberg-Yekutieli method, a less conservative but still stringent statistical approach conceptualizing the rate of type I errors(Benjamini and Hochberg, 1995; Benjamini and Yekutieli, 2005). Partial Eta squared ($\eta_{\text{p}}^{2}$) was also used as an estimate of the effect size for the ANOVA (η^2^_*p*<_ 0.04 small, 0.4 ≤ η^2^_*p*<_ 0.14 medium, 0.14 ≤ $\eta_{\text{p}}^{2}$ large effect size; Cohen, 1988).

Non-inferiority statistics were used to verify the similarity with baseline values (Walker and Nowacki, 2010). For that purpose, 90% confidence intervals were calculated for the differences between baseline values (BDC-1) and values collected after bed-rest (R+0, R+7, R+13, R+28, and R+90) according to Piaggio et al. (2012). Note that for non-inferiority testing, an alpha level of 0.05 corresponds to the 90% confidence interval. The acceptable bounds were determined for each parameter separately, based on the differences observed between single trials assessed during BDC-1 or between trials assessed during BDC-14 and BDC-1. If the results were statistically non-inferior to baseline the respective parameter was marked with a ≈ symbol. One-tailed Pearson's correlation coefficients were calculated for CTRL, between percentage change in the CoF path length and the CCIs for R+0. One-tailed Pearson's correlation coefficients were calculated for the respective percentage changes for R+0 to determine the strength of linear relations between the degradation in posture control, gait and TUG. Statistical tests were executed with SPSS 25.0 (SPSS, Inc., Chicago, IL, USA). Group data is presented as mean value ± standard deviation.

## Results

### Posture control

#### Sway path

The measured parameters associated with equilibrium control remained constant after the 60 days of bed-rest for JUMP, whereas CTRL showed changes ranging between 30 and 105% (Table 1, Figure 2B). This was true for both eyes open and eyes closed conditions. The dominant CoF frequency was non-inferior after bed-rest compared to baseline values in both groups. The ANOVA revealed significant group^*^time interaction effects, with between-group effect sizes ranging from medium to large (Table 1). Non-inferiority statistics showed that most parameters were similar between R+0, R+7, R+13, R+28, and R+90 and BDC-1 in the JUMP group, and that the adaptations observed in the inactive CTRL group were mostly recovered seven to 90 days after re-ambulation.

**Table 1:** Kinematic changes in posture control with eyes open (top) and eyes closed (bottom).

		**Baseline**		**Recovery**					**Statistics**	
	**Group**	**BDC-14**	**BDC-1**	**R+0**	**R+7**	**R+13**	**R+28**	**R+90**	Interaction group^*^time	**Effect size $\eta_{\text{p}}^{2}$**
**POSTURE CONTROL–EYES OPEN**										
CoF velocity	CTRL	1.05 ± 0.25	1	1.90 ± 0.65^*^	1.39 ± 0.20^*^	1.27 ± 0.35^*^	1.09 ± 0.26≈	1.01 ± 0.29≈	*F*_(1, 21)_ = 39.1	0.65
	JUMP	1.03 ± 0.24	1	1.05 ± 0.34≈	0.97 ± 0.28≈	0.96 ± 0.32≈	1.01 ± 0.34≈	0.93 ± 0.26≈	*P < * 0.003
CoF displacement in AP direction	CTRL	1.05 ± 0.31	1	1.50 ± 0.47^*^	1.22 ± 0.25^*^	1.24 ± 0.23^*^	1.10 ± 0.30≈	1.00 ± 0.23≈	*F*_(1, 21)_ = 45.0	0.68
	JUMP	0.97 ± 0.32	1	1.04 ± 0.23≈	1.03 ± 0.30≈	1.06 ± 0.37≈	1.00 ± 0.26≈	0.96 ± 0.22≈	*P < * 0.003
CoF displacement in ML direction	CTRL	1.00 ± 0.13	1	1.64 ± 0.30^*^	1.32 ± 0.37^*^	1.19 ± 0.41≈	1.21 ± 0.28≈	1.05 ± 0.36≈	*F*_(1, 21)_ = 28.9	0.58
	JUMP	1.06 ± 0.28	1	1.04 ± 0.21≈	1.03 ± 0.28≈	1.06 ± 0.31≈	0.94 ± 0.34≈	0.96 ± 0.36≈	*P < * 0.001
90% std. ellipse dimension AP	CTRL	1.01 ± 0.15	1	1.64 ± 0.50^*^	1.32 ± 0.49^*^	1.11 ± 0.47≈	1.22 ± 0.28^*^	1.05 ± 0.30≈	*F*_(1, 21)_ = 31.3	0.60
	JUMP	1.05 ± 0.29	1	1.01 ± 0.22≈	1.04 ± 0.30≈	1.02 ± 0.32≈	1.04 ± 0.29≈	0.88 ± 0.21≈	*P < * 0.001
90% std. ellipse dimension ML	CTRL	1.05 ± 0.31	1	1.56 ± 0.47^*^	1.22 ± 0.25^*^	1.13 ± 0.32≈	1.06 ± 0.33≈	1.00 ± 0.23≈	*F*_(1, 21)_ = 26.8	0.56
	JUMP	0.97 ± 0.26	1	1.04 ± 0.23≈	1.03 ± 0.30≈	1.06 ± 0.37≈	1.08 ± 0.36≈	0.96 ± 0.21≈	*P* = 0.002
Dominant frequency	CTRL	1.04 ± 0.19	1	1.13 ± 0.34	0.89 ± 0.21≈	0.96 ± 0.21≈	1.01 ± 0.35≈	1.04 ± 0.27≈	*F*_(1, 21)_ = 0.7	0.03
	JUMP	1.04 ± 0.28	1	1.01 ± 0.35≈	0.95 ± 0.28≈	1.03 ± 0.35≈	1.04 ± 0.20≈	1.06 ± 0.29≈	*P* = 0.58
**POSTURE CONTROL–EYES CLOSED**										
CoF displacement	CTRL	1.03 ± 0.24	1	2.05 ± 0.55^*^	1.41 ± 0.84≈	1.34 ± 0.79^*^	1.09 ± 0.41≈	1.12 ± 0.32≈	*F*_(1, 21)_ = 6.9	0.25
	JUMP	1.05 ± 0.22	1	1.24 ± 0.36	1.07 ± 0.55	1.10 ± 0.32≈	1.03 ± 0.40≈	0.91 ± 0.33≈	*P* = 0.02
CoF velocity	CTRL	1.07 ± 0.35	1	2.10 ± 0.64^*^	1.27 ± 0.36^*^	1.24 ± 0.46^*^	1.10 ± 0.39	1.19 ± 0.39	*F*_(1, 21)_ = 7.2	0.26
	JUMP	1.09 ± 0.24	1	1.02 ± 0.27≈	1.03 ± 0.32≈	1.04 ± 0.26≈	1.12 ± 0.40	0.91 ± 0.41≈	*P < * 0.001
CoF displacement in AP direction	CTRL	1.03 ± 0.37	1	2.07 ± 0.59^*^	1.24 ± 0.63^*^	1.29 ± 0.66	1.17 ± 0.53	1.05 ± 0.30≈	*F*_(1, 21)_ = 19.2	0.48
	JUMP	1.09 ± 0.24	1	0.99 ± 0.35≈	1.10 ± 0.51≈	1.07 ± 0.37≈	1.02 ± 0.38≈	0.84 ± 0.47≈	*P* = 0.002
CoF displacement in ML direction	CTRL	1.11 ± 0.47	1	2.15 ± 0.45^*^	2.14 ± 0.44^*^	1.53 ± 0.43^*^	1.43 ± 0.35^*^	1.06 ± 0.25≈	*F*_(1, 21)_ = 7.2	0.26
	JUMP	1.12 ± 0.33	1	1.07 ± 0.21≈	1.05 ± 0.48≈	1.10 ± 0.37≈	1.04 ± 0.36≈	0.98 ± 0.48≈	*P* = < 0.001
90% std. ellipse area	CTRL	0.93 ± 0.40	1	2.12 ± 0.98^*^	1.51 ± 1.07^*^	1.39 ± 0.73	1.27 ± 0.89	1.16 ± 0.51≈	*F*_(1, 21)_ = 48.6	0.70
	JUMP	1.05 ± 0.32	1	1.17 ± 0.60≈	0.86 ± 0.27≈	1.13 ± 0.65≈	0.94 ± 0.50≈	0.97 ± 0.36≈	*P < * 0.001
90% std. ellipse dimension AP	CTRL	1.13 ± 0.51	1	2.15 ± 0.98^*^	1.14 ± 0.50≈	1.13 ± 0.48≈	1.03 ± 0.36≈	1.06 ± 0.26≈	*F*_(1, 21)_ = 37.8	0.64
	JUMP	1.12 ± 0.37	1	1.06 ± 0.23≈	1.05 ± 0.51≈	1.17 ± 0.47≈	0.96 ± 0.26≈	0.94 ± 0.49≈	*P < * 0.001
90% std. ellipse dimension ML	CTRL	0.90 ± 0.39	1	2.23 ± 0.68^*^	1.61 ± 0.76^*^	1.29 ± 0.85	1.17 ± 0.62	1.05 ± 0.30≈	*F*_(1, 21)_ = 40.9	0.66
	JUMP	1.07 ± 0.24	1	1.02 ± 0.32≈	1.10 ± 0.51≈	1.06 ± 0.40≈	1.00 ± 0.39≈	0.94 ± 0.39≈	*P < * 0.001
Dominant frequency	CTRL	1.02 ± 0.35	1	0.96 ± 0.29≈	0.85 ± 0.31≈	0.92 ± 0.26≈	1.03 ± 0.24≈	1.01 ± 0.29≈	*F*_(1, 21)_ = 0.4	0.02
	JUMP	1.08 ± 0.35	1	1.02 ± 0.39≈	1.06 ± 0.40≈	0.96 ± 0.36≈	0.95 ± 0.24≈	1.05 ± 0.25≈	*P* = 0.82

Results of the balance test are displayed for the JUMP inactive CTRL for baseline measurements (BDC-14 and BDC-1) and the recovery phase (R+0, R+7, R+13, R+28 and R+90) after bed-rest. Values are normalized to BDC-1. Values are means ± SE.

* *indicates a significant difference compared to baseline. The ≈ symbol non-inferiority compared to baseline. CoF, center of force; ML, medio-lateral; AP, anterior-posterior; std., standard*.

#### Neuromuscular activation–co-contraction

**Figure 4:**
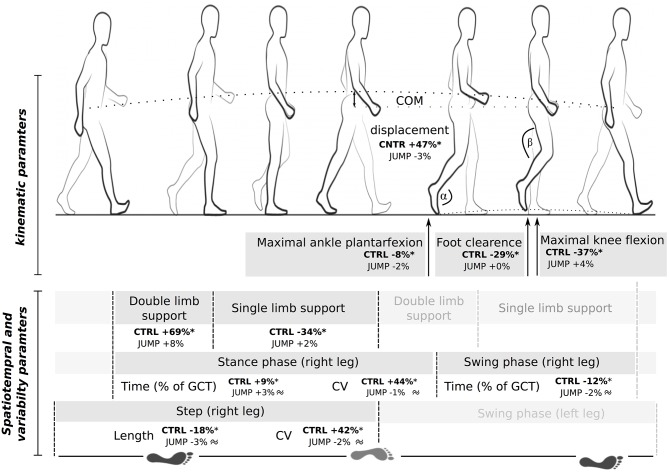
Gait characteristics of the inactive CTRL and JUMP group after bed-rest illustrated for one stride. Changes refer to R+0 normalized to BDC-1. Swing phase, double and single limb support are expressed as %-changes normalized to the gait cycle time (GCT). Values are means ± SE. ^*^ indicates a significant difference and the ≈ symbol non-inferiority compared to baseline. CV, coefficient of variance; GCT, gait cycle time; COM, center of mass.

Non-inferiority statistics showed that the CCIs for SOL_TA, GM_TA, RF_TA, and VM_TA remained constant between R+0, R+7, R+13, R+28, and R+90 and BDC-1 in JUMP, whereas the inactive CTRL showed significantly increased CCIs (Figure 4). Adaptations observed in CTRL were mostly recovered 2 to 4 weeks after the end of bed-rest.

### Gait

Most of measured gait parameters were non-inferior after bed-rest for JUMP compared to baseline values, whereas CTRL showed significant changes in spatiotemporal, kinematic and variance characteristics. This was true for the locomotor tests performed with maximal (Table 2, Figures 2B, 4) and preferred gait speed (Table 3). The ANOVA showed significant group^*^time interaction effects, with between-group effect sizes ranging from medium to large (Tables 1, 2). Plantarflexion during push off, the minimal foot clearance and maximal knee flexion during the swing phase were significantly reduced in CTRL. Concomitantly, gait speed (maximal gait speed *F*_(1, 21)_ = 10.3, *P* = 0.01, $\eta_{\text{p}}^{2}$ = 0.33), step length and cadence were significantly reduced, whereas step time, COM displacement, double limb support (maximal gait speed *F*_(1, 21)_ = 3.3, *P* = 0.06, $\eta_{\text{p}}^{2}$ = 0.14) and single limb support, stance phase and swing phase normalized to total gait cycle time were significantly increased. The CV of step length, step time and stance time was significantly increased after bed-rest. All the deteriorations that were only observed in the inactive CTRL were recovered between seven to 90 days after re-ambulation. Non-inferiority was shown for most of the spatiotemporal, kinematic and variability parameter for R+0, R+7, R+13, R+28, and R+90 compared to baseline values in JUMP, indicating that the gait pattern remained stable throughout bed-rest in the countermeasure group.

**Table 2:** Spatiotemporal, variability and kinematic characteristics in gait with maximal speed.

**Gait - maximal speed**		**Baseline**		**Recovery**					**Statistic**	
	**Group**	**BDC-14**	**BDC-1**	**R+0**	**R+7**	**R+13**	**R+28**	**R+90**	Interaction group^*^time	**Effect size $\eta_{\text{p}}^{2}$**
**SPATIOTEMPORAL PARAMETER**										
Step length	CTRL	0.99 ± 0.08	1	0.82 ± 0.17^*^	0.93 ± 0.13^*^	0.97 ± 0.10≈	1.00 ± 0.07≈	1.01 ± 0.04≈	F_(1, 21)_ = 3.2	0.13
	JUMP	1.01 ± 0.08	1	0.97 ± 0.08≈	0.98 ± 0.08≈	0.99 ± 0.09≈	0.99 ± 0.10≈	0.99 ± 0.08≈	P = 0.04
Step time	CTRL	1.02 ± 0.08	1	1.07 ± 0.12^*^	0.97 ± 0.06≈	0.96 ± 0.07≈	0.98 ± 0.09≈	0.97 ± 0.08≈	F_(1, 21)_ = 0.9	0.04
	JUMP	1.01 ± 0.10	1	1.01 ± 0.11≈	1.01 ± 0.14≈	0.97 ± 0.11≈	0.96 ± 0.14≈	0.98 ± 0.10≈	P = 0.13
Cadence	CTRL	1.00 ± 0.11	1	0.86 ± 0.12^*^	0.89 ± 0.10^*^	0.95 ± 0.13	1.03 ± 0.11≈	1.03 ± 0.09≈	F_(1, 21)_ = 26.8	0.56
	JUMP	1.03 ± 0.12	1	1.03 ± 0.11≈	0.97 ± 0.09≈	0.99 ± 0.08≈	1.04 ± 0.09≈	1.05 ± 0.11≈	P = 0.001
Single support phase (% of GCT)	CTRL	0.99 ± 0.25	1	1.21 ± 0.23≈	1.18 ± 0.20^*^	0.99 ± 0.27≈	1.09 ± 0.31	0.95 ± 0.27≈	F_(1, 21)_ = 33.0	0.61
	JUMP	1.03 ± 0.17	1	1.03 ± 0.16≈	0.98 ± 0.21≈	0.90 ± 0.23	0.91 ± 0.23	0.91 ± 0.21	P = 0.48
Stance phase (% of GCT)	CTRL	1.01 ± 0.03	1	1.09 ± 0.07^*^	1.04 ± 0.04^*^	1.02 ± 0.04	1.01 ± 0.03≈	1.00 ± 0.04≈	F_(1, 21)_ = 2.8	0.12
	JUMP	1.01 ± 0.03	1	1.03 ± 0.02	1.01 ± 0.04≈	1.01 ± 0.03≈	1.04 ± 0.02	0.96 ± 0.06	P = 0.04
Swing phase (% of GCT)	CTRL	0.99 ± 0.04	1	0.88 ± 0.12^*^	0.93 ± 0.11^*^	0.98 ± 0.06≈	0.99 ± 0.05≈	1.00 ± 0.05≈	F_(1, 21)_ = 29.1	0.58
	JUMP	1.01 ± 0.05	1	0.98 ± 0.03≈	1.00 ± 0.04≈	1.01 ± 0.05≈	1.01 ± 0.04≈	1.03 ± 0.05≈	*P* = 0.007
**GAIT VARIABILITY**										
CV step length	CTRL	1.00 ± 0.36	1	1.42 ± 0.37^*^	1.12 ± 0.35≈	0.95 ± 0.25≈	0.98 ± 027≈	0.98 ± 0.31≈	F_(1, 21)_ = 15.2	0.42
	JUMP	0.95 ± 0.31	1	0.98 ± 0.38≈	0.94 ± 0.20≈	0.95 ± 0.28≈	0.98 ± 0.31≈	1.02 ± 0.52≈	*P* = 0.008
CV step time	CTRL	1.07 ± 0.40	1	1.64 ± 0.26^*^	1.17 ± 0.35^*^	1.22 ± 0.35^*^	1.06 ± 0.30≈	1.03 ± 0.25≈	F_(1, 21)_ = 49.4	0.70
	JUMP	0.94 ± 0.21	1	0.97 ± 0.43≈	1.02 ± 0.34≈	0.89 ± 0.30≈	1.03 ± 0.41≈	1.02 ± 0.40≈	*P < * = 0.001
CV stance time	CTRL	1.00 ± 0.49	1	1.44 ± 0.34^*^	1.22 ± 0.34^*^	1.23 ± 0.39^*^	1.23 ± 0.51	1.01 ± 0, 38≈	F_(1, 21)_ = 31.7	0.60
	JUMP	0.98 ± 0.35	1	0.99 ± 0.33≈	1.01 ± 0.52≈	1.06 ± 0.46	1.05 ± 0.40≈	0.95 ± 0.42≈	*P* = 0.001
**GAIT KINEMATICS**										
Peak ankle plantarflexion at push off	CTRL	0.99 ± 0.04	1	0.92 ± 0.08^*^	0.93 ± 0.09^*^	0.97 ± 0.07	0.98 ± 0.05≈	0.99 ± 0.03≈	*F*_(1, 21)_ = 6.1	0.23
	JUMP	1.01 ± 0.05	1	0.98 ± 0.04≈	1.00 ± 0.04≈	0.99 ± 0.40≈	0.99 ± 0.05≈	1.00 ± 0.05≈	*P* = 0.02
Peak knee flexion during swing phase	CTRL	0.96 ± 0.15	1	0.63 ± 0.38^*^	0.74 ± 0.42^*^	0.85 ± 0.27^*^	0.88 ± 0.21	0.91 ± 0.28	*F*_(1, 21)_ = 12.3	0.37
	JUMP	1.07 ± 0.15	1	1.04 ± 0.20≈	1.02 ± 0.25≈	1.01 ± 0.23≈	1.03 ± 0.20≈	0.97 ± 0.15≈	*P* = 0.009
Minimum foot-to-ground clearance	CTRL	1.07 ± 0.22	1	0.71 ± 0.42^*^	0.87 ± 0.35^*^	0.87 ± 0.34^*^	1.00 ± 0.31≈	0.96 ± 0.29≈	*F*_(1, 21)_ = 20.0	0.49
	JUMP	0.99 ± 0.32	1	1.00 ± 0.33≈	0.95 ± 0.36≈	1.04 ± 0.37≈	0.93 ± 0.39≈	1.00 ± 0.34≈	*P* = 0.007
Vertical COM displacement	CTRL	1.08 ± 0.24	1	1.53 ± 0.24^*^	1.51 ± 0.23^*^	1.29 ± 0.19^*^	1.25 ± 0.26^*^	1.18 ± 0.30^*^	*F*_(1, 21)_ = 47.7	0.69
	JUMP	1.00 ± 0.39	1	0.97 ± 0.29≈	0.96 ± 0.33≈	0.94 ± 0.37≈	0.82 ± 0.34≈	0.83 ± 0.37≈	*P < * 0.001

Results for the JUMP and inactive CTRL for the gait test performed with maximal speed are displayed for the baseline measurements (BDC-14 and BDC-1) and the recovery phase (R+0, R+7, R+13, R+28 and R+90) after bed-rest. Values are normalized to BDC-1. Clustered by spatiotemporal parameter, gait variability and gait kinematics, the findings indicate significant differences for R+0 and R+7 compared to baseline. Values are means ± SE.

* *indicates a significant difference compared to baseline. The ≈ symbol non-inferiority compared to baseline. CV, coefficient of variance; GCT, gait cycle time; COM, center of mass*.

**Table 3:** Spatiotemporal, variability and kinematic characteristics in gait with preferred speed.

**Gait– preferred speed**	**Baseline**		**Recovery**					**Statistics**	
	**Group**	**BDC-14**	**BDC-1**	**R**+**0**	**R**+**7**	**R**+**13**	**R**+**28**	**R**+**90**	Interaction group^*^time	**Effect size**$\eta_{\text{p}}^{2}$
**SPATIOTEMPORAL PARAMETER**										
Gait speed	CTRL	0.98 ± 0.13	1	0.78 ± 0.19^*^	0.91 ± 0.13^*^	0.99 ± 0.12≈	0.98 ± 0.12≈	0.98 ± 0.08≈	*F*_(1, 21)_ = 15.5	0.42
	JUMP	0.99 ± 0.19	1	1.02 ± 0.16≈	1.04 ± 0.16≈	1.06 ± 0.16≈	1.05 ± 0.17≈	1.08 ± 0.18≈	*P* = 0.01
Double support time	CTRL	1.02 ± 0.19	1	1.41 ± 0.21^*^	1.18 ± 0.18^*^	1.04 ± 0.18≈	1.04 ± 0.20≈	1.00 ± 0.13≈	*F*_(1, 21)_ = 8.2	0.28
	JUMP	1.02 ± 0.23	1	0.99 ± 0.18≈	0.94 ± 0.17≈	0.97 ± 0.20≈	0.96 ± 0.18≈	0.98 ± 0.20≈	*P* = 0.03
Step length	CTRL	0.96 ± 0.09	1	0.83 ± 0.16^*^	0.94 ± 0.12	1.00 ± 0.09≈	1.01 ± 0.09≈	1.01 ± 0.05≈	*F*_(1, 21)_ = 2.4	0.10
	JUMP	0.97 ± 0.13	1	1.00 ± 0.10≈	1.03 ± 0.10≈	1.03 ± 0.11≈	1.02 ± 0.12≈	1.03 ± 0.05≈	*P* = 0.12
Step time	CTRL	0.99 ± 0.12	1	1.08 ± 0.06	1.04 ± 0.09	1.01 ± 0.06≈	1.03 ± 0.05≈	1.02 ± 0.04≈	*F*_(1, 21)_ = 0.8	0.04
	JUMP	1.03 ± 0.08	1	0.99 ± 0.08≈	0.98 ± 0.06≈	0.97 ± 0.07≈	0.97 ± 0.07≈	0.96 ± 0.08≈	*P* = 0.37
Cadence	CTRL	1.04 ± 0.12	1	0.88 ± 0.13^*^	0.91 ± 0.13^*^	0.96 ± 0.11	1.00 ± 0.12≈	1.01 ± 0.08≈	*F*_(1, 21)_ = 6.9	0.25
	JUMP	1.01 ± 0.08	1	0.99 ± 0.12≈	1.03 ± 0.08≈	0.98 ± 0.12≈	1.02 ± 0.10≈	1.04 ± 0.13	*P* = 0.01
Single support phase (% GCT)	CTRL	1.04 ± 0.15	1	1.25 ± 0.21^*^	1.19 ± 0.24^*^	1.07 ± 0.26≈	1.02 ± 0.23≈	1.00 ± 0.20≈	*F*_(1, 21)_ = 4.5	0.18
	JUMP	0.99 ± 0.20	1	1.00 ± 0.14≈	0.97 ± 0.16≈	0.99 ± 0.15≈	1.00 ± 0.15≈	0.97 ± 0.18≈	*P* = 0.02
Stance phase (% GCT)	CTRL	1.02 ± 0.03	1	1.08 ± 0.05^*^	1.04 ± 0.06	1.00 ± 0.07≈	1.01 ± 0.03≈	1.01 ± 0.02≈	*F*_(1, 21)_ = 3.0	0.13
	JUMP	1.00 ± 0.04	1	1.00 ± 0.02≈	0.99 ± 0.02≈	0.99 ± 0.03≈	1.00 ± 0.03≈	0.99 ± 0.03≈	*P* = 0.07
Swing phase (% GCT)	CTRL	0.97 ± 0.05	1	0.88 ± 0.10^*^	0.94 ± 0.06^*^	1.00 ± 0.10≈	0.98 ± 0.06≈	0.99 ± 0.03≈	*F*_(1, 21)_ = 7.2	0.26
	JUMP	0.99 ± 0.05	1	1.00 ± 0.04≈	1.01 ± 0.04≈	1.01 ± 0.05≈	1.00 ± 0.04≈	1.02 ± 0.05≈	*P* = 0.03
**GAIT VARIABILITY**										
CV step length	CTRL	0.99 ± 0.41	1	1.34 ± 0.36^*^	1.09 ± 0.34	1.03 ± 0.36≈	1.03 ± 0.40≈	1.10 ± 0.43	*F*_(1, 21)_ = 0.9	0.04
	JUMP	1.03 ± 0.49	1	1.00 ± 0.31≈	1.03 ± 0.40≈	0.97 ± 0.46≈	1.04 ± 0.23≈	0.99 ± 0.39≈	*P* = 0.21
CV step time	CTRL	1.04 ± 0.31	1	1.60 ± 0.31^*^	1.21 ± 0.23^*^	1.10 ± 0.23	0.94 ± 0.23≈	1.03 ± 0.19≈	*F*_(1, 21)_ = 27.9	0.57
	JUMP	1.04 ± 0.14	1	1.05 ± 0.21≈	1.03 ± 0.23≈	0.90 ± 0.22≈	0.96 ± 0.24≈	1.06 ± 0.14≈	*P < * 0.001
CV stance time	CTRL	1.04 ± 0.30	1	1.54 ± 0.33^*^	1.25 ± 0.25^*^	0.95 ± 0.34≈	0.98 ± 0.33≈	1.01 ± 0.31≈	*F*_(1, 21)_ = 23.0	0.52
	JUMP	0.98 ± 0.16	1	1.06 ± 0.25	0.94 ± 0.21≈	0.95 ± 0.19≈	0.95 ± 0.36≈	0.96 ± 0.20≈	*P < * 0.001
**GAIT KINEMATICS**										
Peak ankle plantarflexion at push off	CTRL	0.99 ± 0.04	1	0.91 ± 0.06^*^	0.94 ± 0.07^*^	0.96 ± 0.06	0.99 ± 0.05≈	0.99 ± 0.03≈	*F*_(1, 21)_ = 3.7	0.15
	JUMP	1.00 ± 0.04	1	0.98 ± 0.04≈	1.00 ± 0.04≈	1.00 ± 0.04≈	0.99 ± 0.05≈	1.00 ± 0.04≈	*P* = 0.04
Peak knee flexion during swing phase	CTRL	0.99 ± 0.02	1	0.95 ± 0.04^*^	0.98 ± 0.04≈	0.99 ± 0.02≈	1.00 ± 0.02≈	1.00 ± 0.02≈	*F*_(1, 21)_ = 2.9	0.12
	JUMP	1.00 ± 0.02	1	1.01 ± 0.02≈	1.03 ± 0.04≈	1.00 ± 0.02≈	1.00 ± 0.02≈	0.99 ± 0.03≈	*P* = 0.02
Minimum foot-to-ground clearance	CTRL	1.02 ± 0.25	1	0.70 ± 0.38^*^	0.81 ± 0.41^*^	1.01 ± 0.31≈	0.97 ± 0.24≈	0.98 ± 0.33≈	*F*_(1, 21)_ = 3.9	0.16
	JUMP	1.01 ± 0.30	1	1.16 ± 0.22	1.10 ± 0.23	1.05 ± 0.39≈	1.01 ± 0.26≈	1.01 ± 0.49≈	*P* = 0.04
Vertical COM displacement	CTRL	1.06 ± 0.23	1	1.30 ± 0.34^*^	1.18 ± 0.24^*^	1.16 ± 0.17^*^	1.21 ± 0.15^*^	1.14 ± 0.16≈	*F*_(1, 21)_ = 28.1	0.57
	JUMP	0.96 ± 0.25	1	1.01 ± 0.26≈	1.01 ± 0.26≈	1.05 ± 0.26≈	1.02 ± 0.29≈	1.02 ± 0.24≈	*P < * 0.001

Results for the gait test performed at individually preferred speeds are displayed for the JUMP and the inactive CTRL for the baseline measurements (BDC-14 and BDC-1) and the recovery phase (R+0, R+7, R+13, R+28 and R+90) after bed-rest. Values are normalized to BDC-1. Clustered by spatiotemporal parameter, gait variability and gait kinematics the findings indicate significant differences for R+0 and R+7 compared to baseline. Values are means ± SE.

* *indicates a significant difference compared to baseline. The ≈ symbol non-inferiority compared to baseline. CV, coefficient of variance; GCT, gait cycle time; COM, center of mass; Student's t-test*.

### Timed up and go

The time to complete a right and left turn in the TUG test remained constant after the 60 days of bed-rest for JUMP, whereas the CTRL showed significant changes (20–40%; Figure 2B). Values for CTRL returned to baseline 14 days after re-ambulation. The ANOVA revealed significant group^*^time interaction effects with large between-group effect sizes (left turn *F*_(1, 21)_ = 14.9, *P* = 0.002, $\eta_{\text{p}}^{2}$ = 0.42 and right turn *F*_(1, 21)_ = 29.3, *P* < 0.001, $\eta_{\text{p}}^{2}$ = 0.58).

### Chair-rising

Non-inferiority was shown for parameters associated with time and power for R+0, R+7, R+13, R+28, and R+90 compared to baseline values in JUMP, whereas the inactive CTRL showed significant adaptations ranging between 20 and 80% (Figure 2B). The adaptations observed in CTRL were recovered 14 to 28 days after the end of bed-rest. The ANOVA revealed significant group^*^time interaction effects with medium and large between-group effect sizes (time per iteration *F*_(1, 21)_ = 20.1, *P* = 0.01, $\eta_{\text{p}}^{2}$ = 0.49 and maximal power *F*_(1, 21)_ = 7.2, *P* = 0.04, $\eta_{\text{p}}^{2}$ = 0.26).

### Correlations

**Figure 5:**
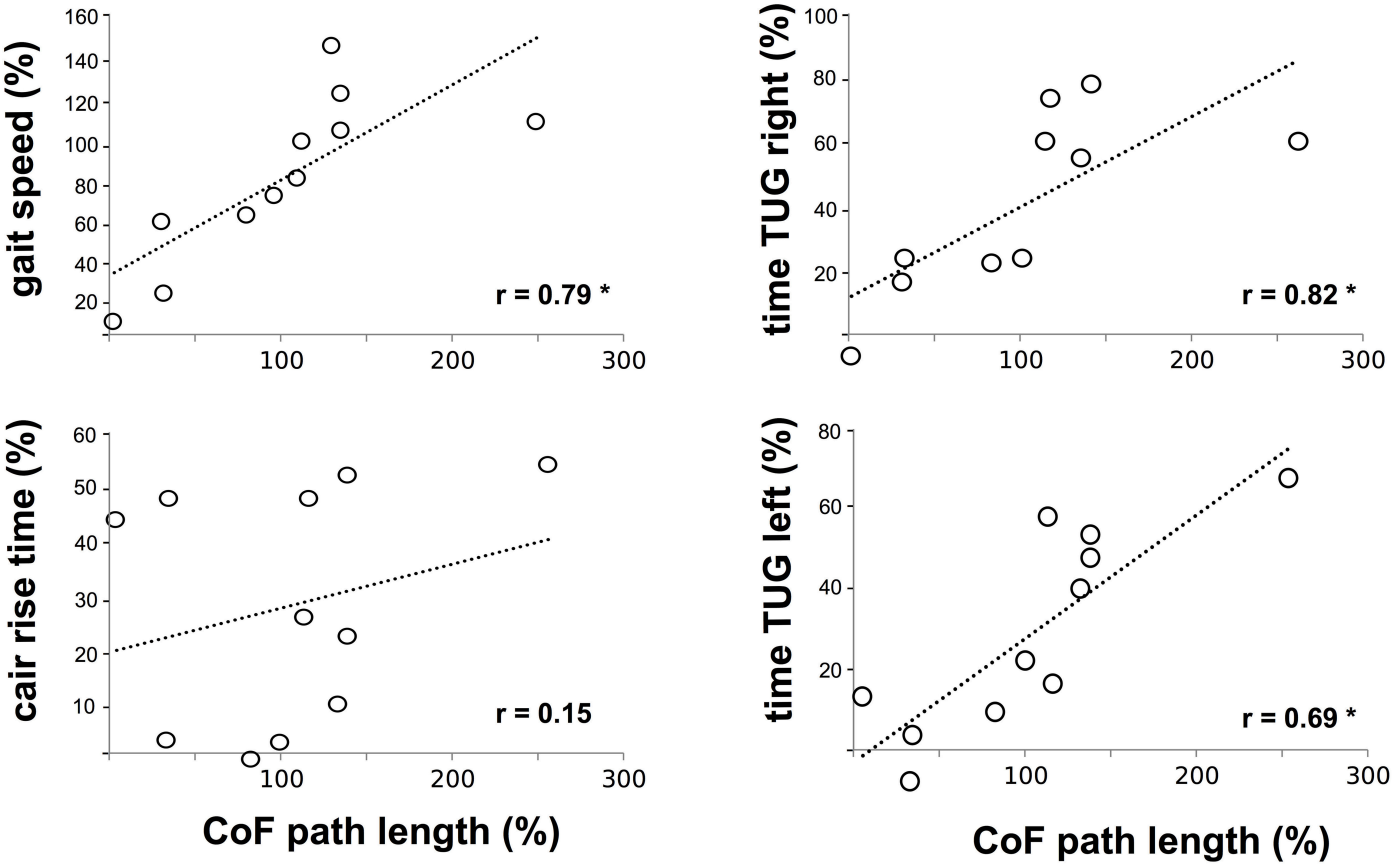
Interrelation between deficits in posture control, gait and functional mobility: Pearson's correlation coefficients among the variables center of force (CoF) path length (abscissa) with the gait speed, chair-rising time (left ordinate) and time to accomplish the Timed Up and Go (TUG) test (right ordinate) for the CTRL calculated for differences between values at R+1 and BDC-1. Findings revealed positive correlations indicating an interrelationship between the decline in posture control and the slowdown in gait and TUG speed. ^*^indicates significant findings.

We detected a significant positive correlation between the CoF sway path and CCI of SOL_TA, GM_TA, VM_BF and RF_BF (Figure 4), indicating that an increased sway path was associated with a higher antagonistic co-contraction in the proximal and distal limb segments. CoF sway path was also positively correlated to the time needed to accomplish the gait test at maximal gait speed and TUG (Figure 5). No significant interrelations were observed for CoF path length and chair-rising time (Figure 5).

## Discussion

This study permits a major insight into countermeasure exercise prescription. The jump exercise was effective in preventing deficits in posture control, gait and functional mobility after 2 months of bed-rest. The jump exercise successfully preserved neural activation patterns involving antagonistic muscles when controlling postural equilibrium. These findings show that a high load, plyometric training program with a short exercise duration can counteract functional degradations in posture and gait control by effectively preventing motor coordination of leg muscles.

### Effect of chronic bed-rest

Complete inactivity during bed-rest, in contrast, led to postural and locomotor deficits and a reduction in functional mobility with a persistence of 2 to 4 weeks after re-ambulation, as confirmed by other studies (Haines, 1974; Dupui et al., 1992; Viguier et al., 2009; Muir et al., 2011). Considering the massive decline in postural equilibrium and its negative impact on gait dynamics, and the time to accomplish TUG predicting 69–82% of its variability, it is not surprising that after bed-rest the inactive control group showed locomotor inconsistencies and abnormalities in muscle coordination far from the normative locomotor values of a healthy human (Kerrigan et al., 1995; Whittle, 1996; Lai et al., 2012; Gouelle and Megrot, 2018).

First, an increased sway path and 90% standard ellipse area was manifested concomitant with an increased co-contraction reflected in simultaneously activated antagonistic muscle groups encompassing the ankle and knee joint. Augmented co-contractions are related to a rigid articular stiffening and have been postulated as a safety strategy to enhance security during single limb support to narrow the risk of falling and injury (Hortobágyi et al., 2009; Nagai et al., 2011; Sayenko et al., 2012) while restricting the ability to react precisely to sudden postural perturbations (Allum et al., 2002; Tucker et al., 2008). It may therefore be assumed that the increased co-contraction is a protective mechanism utilized in difficult postural tasks by individuals suffering from muscle weakness and fragility after chronic bed-rest (Kramer et al., 2017a,b), however, leading to an augmented postural sway (Hortobágyi et al., 2009; Nagai et al., 2011) (Figure 4). The condition with eyes open and closed, and the frontal and sagittal trajectories, were similarly affected by bed-rest. The scope of the finding is seen in its transfer effects extending to dynamic movement: mastering body equilibrium is apparently a fundamental prerequisite for various daily movements and its proper degradation overlaps with complex cyclic movement, as indicated by the correlation between the increased sway path and reduced gait speed or time to accomplish TUG (Figure 5).

Second, the bipedal locomotor pattern of the inactive control group after bed-rest was of pathological significance. Findings were independent of the modalities preferred or maximal gait speed. The gait deficits included spatiotemporal, kinematic and variability adaptations that have been empirically identified as predisposing a person to a greater gait instability (Malatesta et al., 2003) and subsequently to an increased risk of fall (Whittle, 1996; Maki, 1997; Gouelle and Megrot, 2018). With an emphasis on gait anomalies, the findings of the current study are of clinical relevance: (i) a decline in pace characterized by gait speed, step time and length is associated with reduced executive function and performance (Watson et al., 2010); (ii) rhythm changes characterized by cadence, swing stance time, and double and single limb support, are related to higher fall rates (Verghese et al., 2009); (iii) reduced foot-to-ground clearance and knee flexion during the leg swing phase can cause tripping and an increased fall incidence (Lai et al., 2012); and an increased vertical COM excursion caused by a smaller plantarflexion at push off has been linked to energetic inefficiency (Kerrigan et al., 1995). An increase in the spatiotemporal variability domain—which was also observed in our study—has been identified as the best predictor of future falls (Whittle, 1996; Gouelle and Megrot, 2018). The aforementioned gait abnormalities detected after 60 days of bed-rest in the inactive control group are typically known in the elderly population (Hollman et al., 2011) or in patients with neurological disorders (Gouelle and Megrot, 2018).

Functional mobility assessed by TUG and repeated chair-rises is impaired. The time required to accomplish the test was increased after bed-rest for both testing modalities. Chair rises were also executed with reduced peak power, indicting a reduced power-generating capacity and underscoring the results of Kramer et al. (2017b), who similarly demonstrated these degradations for countermovement jumps and ballistic movement.

### Effect of the jump exercise

The impact of the jump exercise during bed-rest was of major significance in the weeks after re-ambulation. Interaction effects demonstrate significant differences between both groups after the end of bed-rest and its benefits equally affect posture control, gait, and functional mobility: while the entire recovery phase is characterized by comparable values over time that differ only marginally to baseline in JUMP, great differences in comparison with the inactive CTRL were established for R+0 and R+7 in the tests, including posture control, gait and functional mobility. Concomitantly, neuromuscular control of the skeletal muscle with an emphasis on antagonistic coordination could be preserved throughout the high-intensity jump exercise performed during bed-rest. Other bed-rest studies validating countermeasures such as strength training (Haines, 1974; Kouzaki et al., 2007), flywheel (Viguier et al., 2009), and treadmill (Macaulay et al., 2016), lower-body negative-pressure (Dupui et al., 1992), mechanical stimulation (Muir et al., 2011) or centrifugation (Vernikos et al., 1996), found plyometric jump training to be advantageous compared to past alternatives. Despite differing HDT periods ranging from 5 days to 12 weeks, none of these countermeasures succeeded in entirely preserving gait, posture and functional mobility during bed-rest (Haines, 1974; Dupui et al., 1992; Vernikos et al., 1996; Kouzaki et al., 2007; Macaulay et al., 2016) despite those that permitted daily upright stance, and used standing and walking as exercise modes (Mulder et al., 2014).

A reasonable explanation as to why the countermeasure proved advantageous above other interventions applied during long-term bed-rest (Dupui et al., 1992; Koppelmans et al., 2015; Paloski et al., 2017), with significant treatment effects beyond the actual exercise mode (horizontal jumps), may involve the specific attributes of the countermeasure associated with the preservation of muscle mass and function (Kramer et al., 2017a,b). First, jumps are whole-body movements, which have been shown to elicit larger performance improvements in the lower extremities than segmental single-articular movements (Blackburn and Morrissey, 1998; Stone et al., 2002). Second, jumping is an exercise mode where each repetition requires maximal effort, resulting in exceedingly high forces which are multiples of those occurring during common physical activity such as cycling, stepping or strength training (Komi, 1984). To achieve these peak forces, agonistic muscles need to be contracted entirely while antagonists should be inhibited (Kellis et al., 2003) to reduce co-contraction. Third, jumping relies on the stretch-shortening cycle, which is a natural type of muscle action found in everyday activities such as running, walking and skipping (Taube et al., 2012). An overlap with locomotor movement such as gait and TUG, including the particular neural pattern of synergists and antagonists, may have caused positive effects in the locomotor and mobility test established in our study.

Areas of application for JUMP may range from space-related operations for Astronauts during long-term space missions (Kramer et al., 2017a) to the interface of geriatrics (McGregor et al., 2014), clinical orthopedics (Bugbee et al., 2016), and neurodegeneration (Azizi et al., 2017). Importantly, JUMP can be executed with a wide range of impact loads induced by the sledge's acceleration profile as the equivalent to gravitation within boundaries of 0.5 g up to 1.3 g (Kramer et al., 2017b). Thus, the trainings intensity can be adjusted to the individuals' health and fitness status which allows many different application options to counteract deconditioning induced by inactivity.

### Recovery after bed rest

Long-term recovery after 60 days bed-rest differed between JUMP and CTRL as indicated by group^*^time interaction effects. The countermeasure successfully maintained the neuromuscular system's ability to safely control postural equilibrium and perform complex locomotor movements, and thus, values were mostly comparable to baseline at any point in the recovery phase (Figure 1). In contrast, analysis of the follow-up performance measurements in CTRL during the recovery period showed that even though the decline in functional mobility, posture and gait control was high, detrimental effects were reversible and the recovery was almost complete 1 month after re-ambulation. This is valid for a bed-rest period of 2 months, but would certainly differ for shorter or longer periods (Pavy-Le Traon et al., 2007). Importantly, the range and timescale of the neuromuscular parameters were almost identical to those manifested for the functional deficits. Coupled with positive correlations for changes in posture control with antagonistic co-contraction of the limb musculature, these findings support our hypothesis that neuromuscular mechanisms may underlie functional degradations in addition to the loss in muscle mass (Kramer et al., 2017a,b), and may explain the potential benefit of the selected countermeasure. It is worth discussing the delayed recovery characteristics of the lateral CoF displacement and COM trajectories in gait, which take up to 3 months to return to pre-bed-rest levels. Both factors are associated with the control of the trunk which is the body segment with the largest mass and highest moment of inertia, and are crucial for COM reposition above the base of support (Stapley et al., 1999; You et al., 2001). It is therefore assumed that disregarding the different test paradigms individuals experience great and prolonged difficulties in mastering trunk movement so as to diminish disturbing torques and destabilizing the human body (Kerrigan et al., 1995; Stapley et al., 1999) after bed-rest.

### Limitations

For a conclusive statement, it is crucial to consider the limitations of the study. Two aspects are of substantial importance; the first deals with the study design, and the second with the methodological approach. Although this study provides scientific evidence for exercise prescription in bed-ridden individuals, the application and transferability of the protocol to the clinical context, nursing homes or in spaceflight needs to be specified. The sample size was restricted to 24 young and healthy individuals due to restrained temporal, logistical and financial resources. The limited number of participants may have resulted in non-significant findings regarding the effect of the exercise protocol. Further, in an effort to reduce inter-subject variability, only the male gender was considered for participation in this bed-rest study. Therefore, with reference to Viguier et al. (2009), we are therefore not certain whether our findings are applicable to women or to people with illness suffering from significant health impairments. The methodology used electromyograms of the relevant leg muscles normalized to pMVCs in order to control for changes in fluid shift, skin impedance, electrode positioning or lean or fat mass that would have interfered with changes in muscle activation in response to physical activity and jump training. pMVCs were executed manually in less controlled conditions than recommended in the current literature, however, and thus they should be seen as a limitation (Burden, 2010).

## Conclusion

The plyometric jump exercise has been justified as a successful and time-efficient countermeasure to prevent the detrimental effects of physical deconditioning during 2 months of bed-rest. It effectively preserved the neuromuscular system's ability, in healthy men, to safely control postural stability, and perform complex locomotor movements, including bipedal gait at maximal speed up to complex locomotor technics with turns and rises. It is noteworthy that positive effects were manifested in the aforementioned paradigms despite the distinct differences between the components of the test battery and the actual jump exercise. We therefore assume that the preservation of muscle mass and function achieved through the countermeasure is sufficient to trigger side effects, with an overlap with the control of gait and posture. We expect that the outcomes of the present study are of major relevance in various scenarios, including Astronauts (Kramer et al., 2017a), orthopedic and neurodegenerative patients (Azizi et al., 2017), the elderly (McGregor et al., 2014) and sedentary populations (Pavy-Le Traon et al., 2007) suffering from physical deconditioning due to chronic inactivity.

## Author contributions

RR, KF, AK, JK, MG, DB, DF, AG, and GA: Conceptualization. RR and AG: Funding acquisition. RR, KF, AK, and JK: Investigation. RR, KF, AK, JK, MG, DB, DF, AG. GA: Methodology. RR, KF, AK, and AG: Project administration. RR, KF, AK, JK, MG, DB, DF, AG, and GA: Resources. RR and AK: Software. AG, AK, and GA: Supervision. RR: Writing of the original draft. RR: Figure and tables. RR, KF, AK, MG, DB, AG. GA: Review and editing.

### Conflict of interest statement

The authors declare that the research was conducted in the absence of any commercial or financial relationships that could be construed as a potential conflict of interest.
